# Effect of Hematopoietic Stem Cell Transplantation and Post-Transplantation Cyclophosphamide on the Microglia Phenotype in Rats with Experimental Allergic Encephalomyelitis

**DOI:** 10.1007/s00005-023-00675-y

**Published:** 2023-03-24

**Authors:** Kaja Kasarełło, Martyna Seta, Dorota Sulejczak, Emilian Snarski, Agnieszka Cudnoch-Jędrzejewska

**Affiliations:** 1 Chair and Department of Experimental and Clinical Physiology, Centre for Preclinical Research, Medical University of Warsaw, Warsaw, Poland; 2 Department of Experimental Pharmacology, Mossakowski Medical Research Institute, Polish Academy of Sciences, Warsaw, Poland

**Keywords:** Microglia, Cyclophosphamide, Hematopoietic stem cells, Multiple sclerosis, Experimental allergic encephalomyelitis

## Abstract

Microglia are the resident immune cells of the central nervous system, playing a role in the inflammatory process development and resolution, presenting two main phenotypes, pro-inflammatory M1, and anti-inflammatory M2. Therapies affecting the microglia phenotype may be beneficial in treating inflammatory neurodegenerative diseases. In our experiments, we used the animal multiple sclerosis model, experimental allergic encephalomyelitis (EAE). Rats were treated during the pre- or symptomatic phase of the disease with cyclophosphamide, followed by hematopoietic stem cell transplantation, and with/without post-transplantation cyclophosphamide. Our study aimed to analyze the microglia phenotype in animals subjected to this treatment. The number of M1 cells in the spinal cord, and inducible nitric oxide synthase (iNOS) levels in the brain were similar in all experimental groups. The differences were observed in M2 cells number and arginase 1 (Arg1) levels, which were decreased in EAE animals, and increased after treatment in the symptomatic phase of EAE, and in the pre-symptomatic phase, but only with post-transplantation cyclophosphamide. Analysis of gene expression in the brain showed decreased *iNOS* expression in EAE animals treated in the symptomatic phase of EAE and no differences in *Arg1* expression. Results indicate that treatment applied to experimental animals influences the microglia phenotype, promoting differentiation towards M2 cells.

## Introduction

Microglia are the immune cells resident in the central nervous system (CNS), which role is the protection of surrounding tissue against pathogens, as well as the removal of dead cells. Microglia are activated by pathogens, pathogenic substances, or mediators of inflammation released by immune cells infiltrating the CNS through the opened blood–brain barrier (Chu et al. 2018). Microglia participate in the development of an inflammatory reaction, mainly due to the release of the pro-inflammatory cytokines and chemokines, eventually leading to the removal of the pathogen. Microglia has also the ability to perform antigen presentation. It is already known, that microglia besides the classic, pro-inflammatory phenotype (called M1) can differentiate into cells of the anti-inflammatory phenotype (called M2), and such polarization occurs under influence of the anti-inflammatory cytokines. M2 microglia are responsible for quenching the inflammatory reaction by releasing the anti-inflammatory cytokines, mainly interleukin (IL)-10 and transforming growth factor β, and more importantly, the repair of the surrounding tissue which is supported by the release of neurotrophic factors such as brain-derived neurotrophic factor, vascular endothelial growth factor or epidermal growth factor (Bogie et al. 2014; Chu et al. 2018, 2019; Subramaniam and Federoff 2017).

Chronic inflammation induced by pathological processes ongoing in the CNS, leading to secondary destruction of surrounding tissue, often accompanies neurodegenerative diseases such as multiple sclerosis (MS). Microglia, as the resident immune cells in the CNS, mediate the inflammatory reaction during the disease course (Nowacki et al. 2019). In MS, activated microglia are found not only in active lesions but also in chronic ones (Voet et al. 2019). Furthermore, activated microglia are found in the part of the not-inflamed white matter of MS patients (Zrzavy et al. 2017). M1 cells are predominant during the disease relapse, while during remission the polarization towards M2 occurs (Chu et al. 2018). In animals with an evoked animal model of MS (experimental allergic encephalomyelitis, EAE), microglia were shown to be the first antigen-presenting cells (APCs) for infiltrating autoreactive T cells in the initial phase of CNS inflammation (Sosa et al. 2013). Upon activation, microglia increase the expression of MHC and costimulatory molecules (Brendecke and Prinz 2015).

One of the lately approved human therapy for MS is autologous hematopoietic stem cell transplantation (AHSCT) following administration of the immuno- or myeloablative agent, such as cyclophosphamide (Cy). This method assumes the elimination of autoreactive to myelin antigens lymphocyte clones, which are the potential inducers of immune reactions in CNS (Atkins and Freedman 2013; Burman et al. 2018; Cohen et al. 2019; Sharrack et al. 2020). Recently, we tested the effect of such therapy in rats with an animal model of MS – EAE evoked. We showed that applied treatment was efficient in decreasing the clinical symptoms of EAE observed in rats, decreased the number of days with present clinical symptoms, and also reduced the inflammatory infiltrations in the spinal cord. Furthermore, we extended the study with novel variants of therapy involving the administration of an additional, low-dose of Cy after AHSCT what noticeably boosted the effectiveness of the therapy (Kasarełło et al. 2021). The rationale for using the additional low-dose of Cy was to eliminate the autoreactive lymphocytes possibly present in the hematopoietic stem cell graft.

We assumed that observed decreased infiltrations into the CNS may result in reduced levels of inflammatory mediators and may further influence microglia activation and/or differentiation. Consequently, there is a question raising, if such therapy affects the microglia activation and/or differentiation, what may be the part of the mechanism leading to disease amelioration.

The presented experiments are the continuation of our previous studies conducted using the animals with EAE evoked, which results are described in the article of Kasarełło et al. (2021). Current research aims to analyze the mechanism underlying the previously observed changes in the disease course in rats.

## Materials and Methods

This study is the continuation of research published by Kasarełło et al. (2021). The experiments were conducted as described previously. Below, we present the scheme of animal treatment.

### Animals

All procedures were approved by the First Local Ethical Committee in Warsaw (Act No. 494/2018) and Second Local Ethical Committee in Warsaw (Act No. WAW2/129/2018). The regulations and guidelines on the use and care of laboratory animals of the EU Directive 2010/63/EU were followed for the animal experiments. Study details complied with ARRIVE guidelines. Female Lewis rats, approx. 12-week-old were achieved from the Mossakowski Medical Research Centre breeding facility (Warsaw, Poland) and were randomly divided into seven experimental groups and subjected to procedures described below.

#### Treatment Protocols

EAE was induced at day 0 (0 DPI – day post immunization) as described before (Kasarełło et al. 2021). The onset of clinical symptoms in this EAE model was observed at 10–11 DPI. Treatment was started either in the pre-symptomatic (I), or symptomatic (II) phase of the EAE.

Animals were injected with a high-dose of Cy (125 mg/kg, Baxter) 1 day before AHSCT, which was performed by injecting the cell suspension (20 × 10^6^ cells in 0.5 ml PBS) into the tail vein of rats. The method of hematopoietic stem cell collection was described earlier (Kasarełło et al. 2021). Three and 4 days after AHSCT part of the animals received an additional low-dose of Cy (20 mg/kg).

In the first (I) protocol high dose of Cy was given at 6 DPI, AHSCT at 7 DPI, low-dose of Cy at 11 and 12 DPI. In the second (II) protocol high dose of Cy was given at 13 DPI, AHSCT at 14 DPI, low-dose of Cy at 17 and 18 DPI. The time schedule of the animals’ treatment is shown in Table 1.

**Table 1:** Time schedule of the experiment

	First (I) time schedule	DPI	Second (II) time schedule
Presymptomatic phase of EAE	EAE induction	0	EAE induction
		1	
		2	
		3	
		4	
		5	
	High-dose Cy	6	
	HSCT	7	
		8	
		9	
	Low-dose Cy	10	
	Low-dose Cy	11	
		12	
Symptomatic phase of EAE		13	High-dose Cy
		14	HSCT
		15	
		16	
		17	Low-dose Cy
		18	Low-dose Cy
		19	
		20	
	Euthanasia + tissue collection	21	Euthanasia + tissue collection

*HSCT* hematopoietic stem cell transplantation

Not-treated healthy animals, healthy animals with treatment applied, and not-treated rats with EAE evoked served as control.

### Analysis of Selected Microglia Phenotype Markers

#### Tissue Collection

Animals were sacrificed at 21 DPI, after the resolution of the clinical symptoms. Rats were subjected to anesthesia (intraperitoneal injection of 100 mg/kg ketamine with 10 mg/kg xylazine) and decapitated. Brains and spinal cords (cervical and thoracal segments) were collected. One brain hemisphere was further used for protein levels analysis and the second for mRNA expression analysis. Brains were frozen directly in liquid nitrogen, and spinal cords were fixed in the 4% formaldehyde for 24 h and saturated with sucrose through immersing in 10, 20, and 30% (w/v) sucrose solutions in PBS. Tissues were frozen and stored at − 80 °C.

To investigate the microglia phenotype, we performed the immunofluorescent analysis of spinal cord sections using confocal microscopy and analysis of gene expression (real-time PCR) as well as protein levels of microglia markers (ELISA). For the general detection of microglia, we used ionized calcium-binding adapter molecule 1 (Iba1, microglia/macrophage marker) and inducible nitric oxide synthase (iNOS) and arginase 1 (Arg1) were employed as markers of M1 and M2 subtypes of microglia (respectively).

#### Histopathological and Immunofluorescent Analysis

Frozen spinal cords, embedded with tissue freezing medium (Leica, Germany) were cut into Sections (20 μm) using a cryostat (Leica, Germany) and mounted to salinized glass microscope slides.

Histopathological analysis was performed to assess the presence of inflammatory infiltrations in the spinal cord. Tissue slices were dried at room temperature, and subjected to hematoxylin and eosin (H + E) staining according to the standard procedure. Slices were covered with DPX Mountant for histology (Sigma Aldrich, Germany) and analyzed and captured using a light microscope (Nikon, Japan) with a CDC camera (Nikon, Japan). Figures presenting histopathological analysis were prepared using CorelDRAW Graphics Suite (RRID:SCR_014235).

Prior to the immunolabelling procedure for immunofluorescent analysis, sections were dried, fixed with 4% PFA for 30 min, and for 1 h permeabilized and blocked with a mixture of 0.25% Triton X-100 (Sigma Aldrich, Germany), 10% donkey serum (GeneTex, USA) and 1% BSA (Sigma Aldrich, Germany) in PBS. Primary antibodies were applied overnight at 4 °C: Iba1 (Santa Cruz, sc-32725, RRID:AB_667733; 1:200); iNOS (Santa Cruz, sc-7271, RRID:AB_627810; 1:200), and Arg1 (Santa Cruz, sc-271430, RRID:AB_10648473; 1:50). Next, sections were washed with PBS, and secondary antibodies were applied for 1 h at room temperature: Goat anti-Mouse IgG2b Alexa Fluor 546 (Thermo Fisher Scientific, A-21143, RRID:AB_253577; 1:500) and Goat anti-Mouse IgG1 Alexa Fluor 488 (Thermo Fisher Scientific, A-21121, RRID:AB_2535764; 1:500). Cell nuclei were stained with bisbenzymide (Hoechst 33,342, Life Technologies, USA) for 20 min at room temperature. The negative secondary antibodies control was performed to eliminate the false positive results. Zeiss Confocal Laser Microscope LSM 710 (Zeiss LSM 710, Carl Zeiss, Oberkochen, Germany) with a coherent multiphoton unit was used to obtain the images. Figures presenting immunofluorescent analysis were prepared using CorelDRAW Graphics Suite (RRID:SCR_014235).

The number of double-positive, Iba1^+^/iNOS^+^, and Iba1^+^/Arg1^+^ cells were counted in the cervical and thoracal spinal cord sections obtained from rats of each experimental group. Figures presenting the analysis of cell number were prepared using GraphPad Prism software (RRID:SCR_002798).

#### Analysis of Arg1 and iNOS Levels in CNS

Arg1 and iNOS levels were analyzed in one hemisphere of the brains isolated from experimental animals. Brains were homogenized in ice-cold PBS at 50 Hz for 5 min (TissueLyser, Qiagen, Germany), frozen, thawed twice for cell membrane rupture, and centrifuged (10,000 rpm, 10 min, 4 °C). Supernatants were collected. The analysis was performed using Arginase 1 ELISA kit (EIAab,) and Nitric oxide synthase, inducible, ELISA Kit (EIAab) according to the manufacturer’s protocols. Microplates were read using a microplate reader (Microplate Reader, Bio-Rad, USA), and the absorbance (optical density – OD value) at a wavelength of 450 nm was determined. Figures presenting results from ELISA analysis were prepared using GraphPad Prism software (RRID:SCR_002798).

#### Analysis of *Arg1* and *iNOS* Expression in CNS

From the other hemisphere of previously frozen brains, initially chopped, portions weighing 100 µg were collected and then homogenized with Qiazol (QIAzol Lysis Reagent, Qiagen, Germany). Then, total RNA was isolated with RNeasy Lipid Tissue Mini Kit (Qiagen, Germany) according to the manufacturer’s protocol. The concentration and quality of the isolated RNA were measured with a SmartSpec Plus Spectrophotometer (Bio-Rad, USA). The expression level of the genes of interest was assessed using the multiplex real-time PCR method, with TaqMan Gene Expression RNA to CT one-step kit in a ViiA 7 Real-Time PCR System (Applied Biosystems, USA). The following probes were used: iNos2 (Rn00561646_m1) as a marker of M1 phenotype microglia, Arg1 (Rn00691090_m1) as a marker of M2 phenotype microglia, and Gapdh (Rn01775763) as a housekeeping gene. Delta CT method was used to compare gene expression. Figures presenting the analyzed gene expression analysis were prepared using GraphPad Prism software (RRID:SCR_002798).

### Statistical Analysis

Shapiro–Wilk test was used for the distribution of continuous variables analysis. The mean and the standard deviation for normally distributed variables were reported, otherwise, the median and the 25th and 75th percentile (Q1 and Q3) were calculated. The normally distributed continuous variables were compared with the one-way ANOVA test, otherwise, the Kruskal–Wallis test was used. The significance level was set at 0.05. Analysis were performed using GraphPad Prism software (RRID:SCR_002798).

## Results

### Histopathological and Immunofluorescent Analysis

**Fig. 1:**
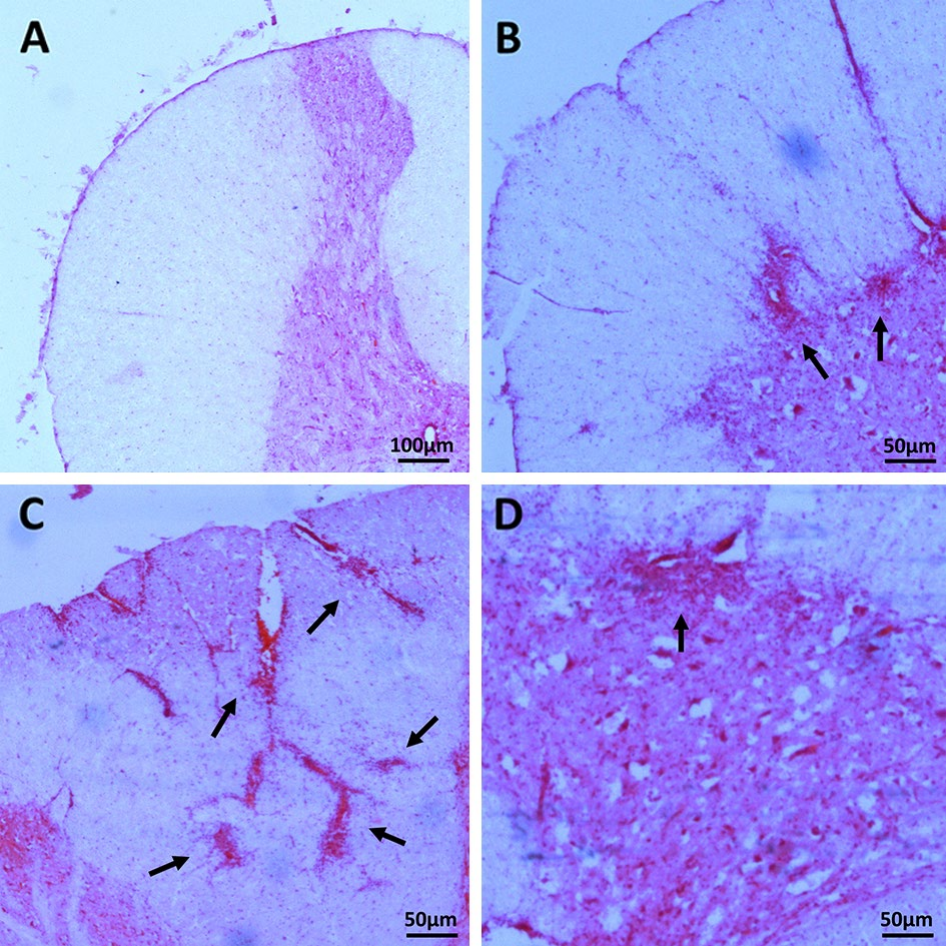
H&E staining of representative spinal cord sections. A dorsal horn without inflammation of control animal without EAE evoked (**A**). Inflammatory infiltration (arrows) was detected at the border of the white and gray matter (**B**), in the white matter of the dorsal spinal cord (**C**), and in the gray matter (**D**), of animals with EAE evoked

Spinal cords slices stained with hematoxylin and eosin were analyzed using a light microscope, to assess the presence of inflammatory infiltrations. We detected the signs of infiltration in the cervical and thoracal spinal cord from animals with EAE evoked, both non-treated (EAE) and treated (EAE Cy HSCT, EAE Cy HSCT Cy, EAE Cy HSCT II, EAE Cy HSCT Cy II) (Fig. 1B–D). Infiltrations were present both in the white and gray matter of the spinal cords, but the abundance of inflammatory cells in the gray matter was lower. There was no visible inflammatory infiltration in the spinal cords obtained from healthy animals, without EAE evoked (NT, Cy HSCT Cy) (Fig. 1A).

The number of M1 and M2 cells was evaluated on the whole surface of the cervical and thoracal spinal cord sections subjected to immunolabelling and analyzed using confocal microscopy. Iba1^+^/iNOS^+^ cells were marked as M1, and Iba1^+^/Arg1^+^ cells were marked as M2 cells.

**Fig. 2:**
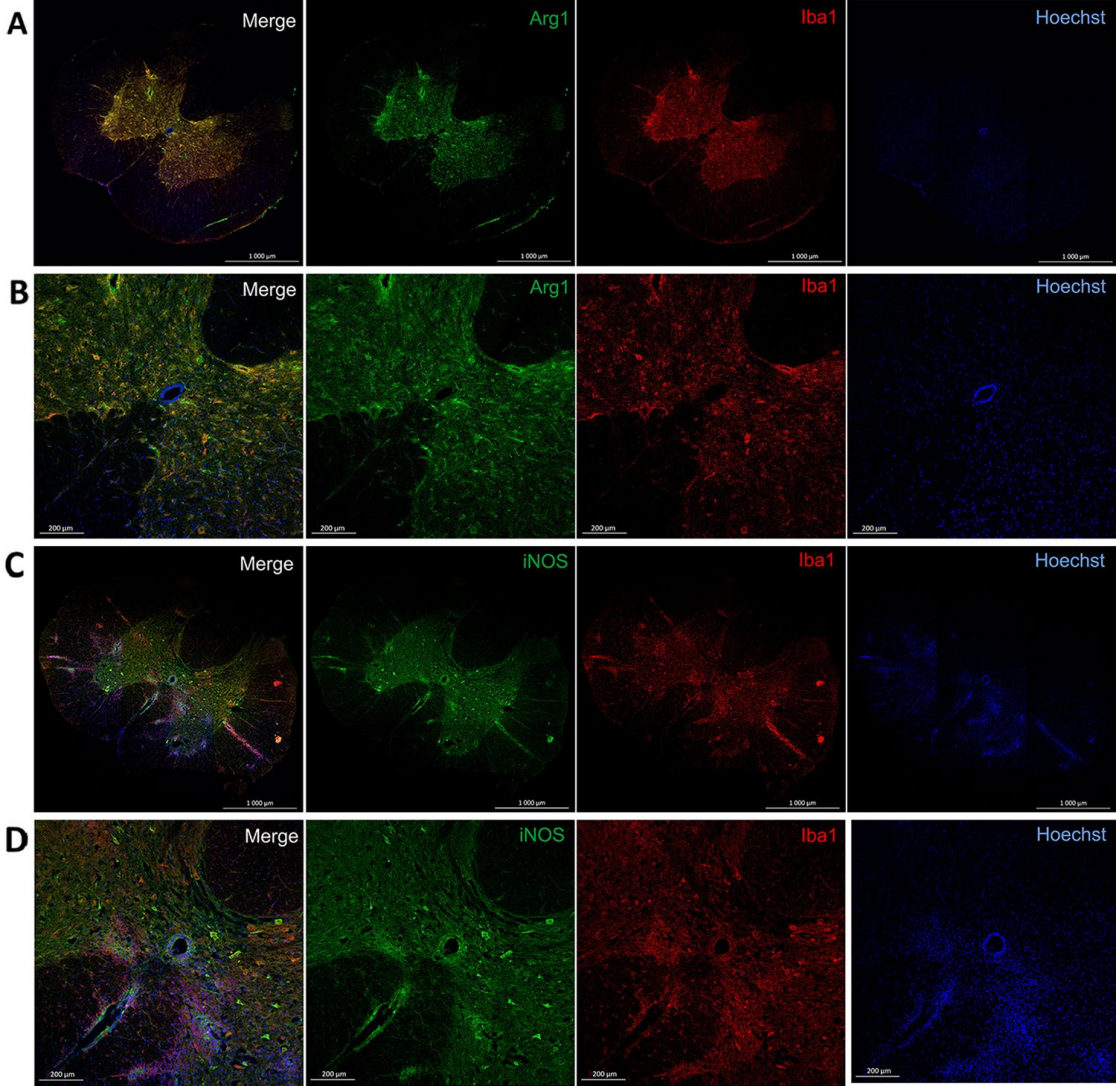
Representative spinal cord sections. Immunolabelling for Iba1/Arg1/Hoechst (**A**, **B**), and Iba1/iNOS/Hoechst (**C**, **D**). Tilescans of whole spinal cord sections (**A**, **C**), images of the central part of spinal cord sections (**B**, **D**). Iba1–microglia marker, Arg1–M2 microglia marker, iNOS–M1 microglia marker, Hoechst–dye visualizing nuclei

Double-positive Iba1^+^/iNOS^+^ and Iba1^+^/Arg1^+^ cells were located mostly in the gray matter of the spinal cord, as well as in inflammatory infiltrations present in white and gray matter (Fig. 2).

**Fig. 3:**
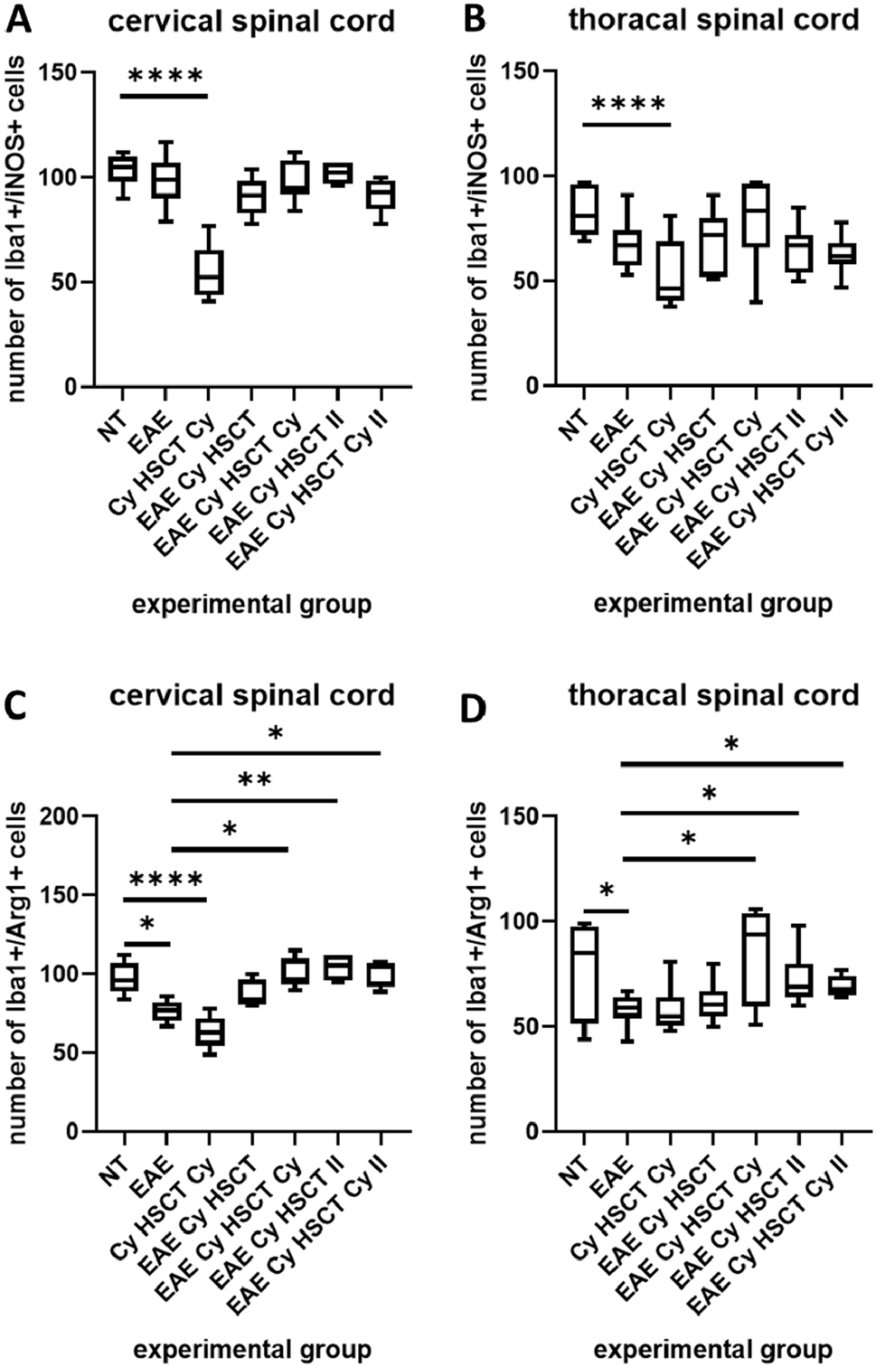
Number of double-positive cells Iba1^+^/iNOS^+^ (M1 microglia marker) in the cervical (**A**) and thoracal (**B**) spinal cord sections, and of Iba1^+^/Arg1^+^ (M2 microglia marker) in the cervical (**C**) and thoracal (**D**) spinal cord sections. Box plot presenting the median (horizontal line), the box covers values from the first to the third quartile and whiskers from the quartiles to the minimum and maximum values. *n* = 5 (EAE Cy HSCT, EAE Cy HSCT Cy, EAE Cy HSCT II, EAE Cy HSCT Cy II), *n* = 6 (NT, Cy HSCT Cy), *n* = 10 (EAE). *p < 0.05; **p < 0.01, ****p < 0.0001

The number of double-positive cells (Iba1^+^/iNOS^+^ and Iba1^+^/Arg1^+^) in the cervical and thoracal spinal cord sections was counted. Both in the cervical (Fig. 3A), and thoracal (Fig. 3B) spinal cord sections number of double-positive Iba1^+^/iNOS^+^ cells was similar, with no differences between experimental groups, except for healthy treated animals (Cy HSCT Cy), where the number of Iba1^+^/iNOS^+^ double-positive cells was considerably lower than in healthy, non-treated (NT) rats.

The changes in the number of co-stained, Iba1^+^/Arg1^+^ cells were similar both in the cervical (Fig. 3C) and thoracal (Fig. 3D) spinal cord. Both in cervical and thoracal sections there was a considerably decreased number of Iba1^+^/Arg1^+^ double-positive cells in EAE non-treated rats (EAE) in comparison to healthy non-treated (NT) rats. In control treated rats (Cy HSCT Cy) there was a substantially lower number of Iba1^+^/Arg1^+^ double-positive cells than in non-treated (NT) rats in cervical spinal cord sections, while in thoracal spinal cord sections, only the tendency was noted. In both cervical and thoracal spinal cord sections, it was shown, that in comparison to EAE non-treated rats (EAE), the number of double-positive Iba1^+^/Arg1^+^ cells was higher in all groups of EAE treated rats (EAE Cy HSCT Cy, EAE Cy HSCT II, EAE Cy HSCT Cy II) except for EAE Cy HSCT group.

### iNOS and Arg1 Levels

**Fig. 4:**
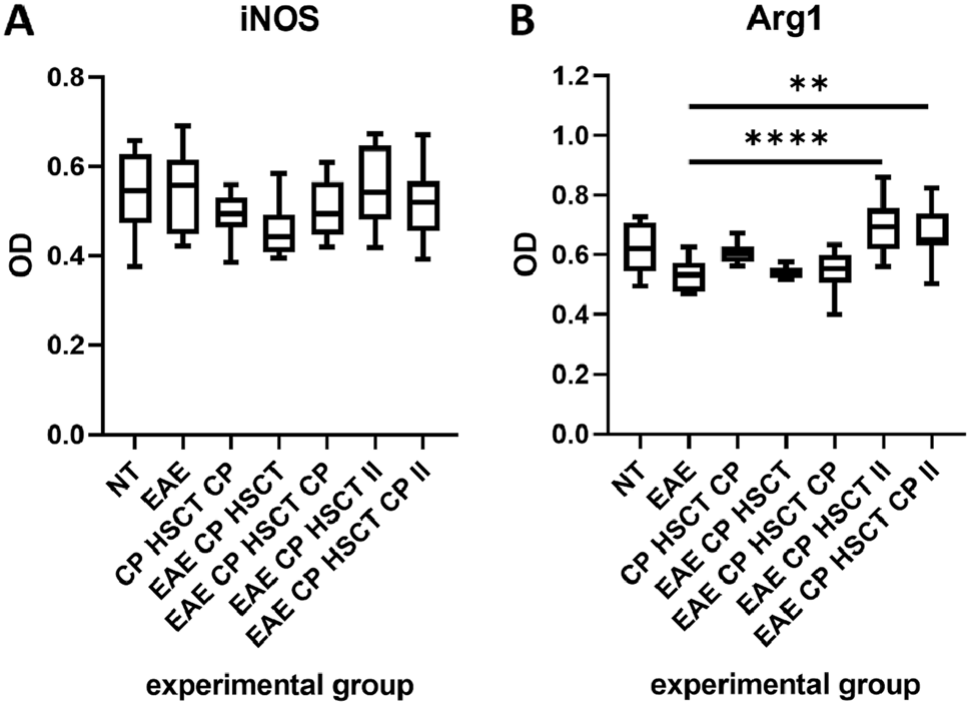
iNOS (**A**) and Arg1 (**B**) levels in brain homogenates of rats. Box plot presenting the median (horizontal line), the box covers values from the first to the third quartile and whiskers from the quartiles to the minimum and maximum values. *n* = 5 (EAE Cy HSCT, EAE Cy HSCT Cy, EAE Cy HSCT II, EAE Cy HSCT Cy II), *n* = 6 (NT, EAE, Cy HSCT Cy). *OD* *value* optical density. **p < 0.01; ****p < 0.0001

Levels of iNOS and Arg1 were measured in brain homogenates of rats. There was no substantial difference observed in iNOS levels between the experimental groups of animals (Fig. 4A). Arg1 levels (Fig. 4B) were considerably increased in EAE animals treated during the symptomatic phase of EAE (EAE Cy HSCT II, EAE Cy HSCT Cy II) in comparison to EAE non-treated (EAE) animals. Levels of Arg1 in the other groups were alike.

### *iNOS* and *Arg1* Expression

**Fig. 5:**
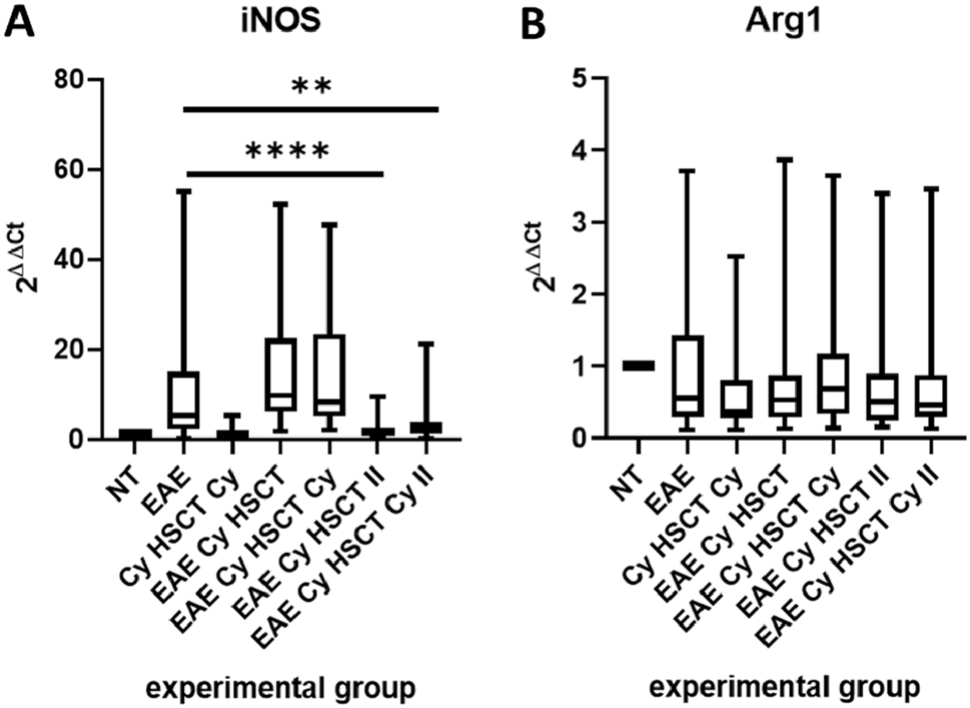
*iNOS* (**A**) and *Arg1* (**B**) expression (2^ΔΔCt^) in brain cell mixture of rats. Box plot presenting the median (horizontal line), the box covers values from the first to the third quartile and whiskers from the quartiles to the minimum and maximum values. *n* = 5 (EAE Cy HSCT, EAE Cy HSCT Cy, EAE Cy HSCT II, EAE Cy HSCT Cy II), *n* = 6 (NT, Cy HSCT Cy), *n* = 10 (EAE). **p < 0.01; ****p < 0.0001

*iNOS* and *Arg1* expression were evaluated in brain homogenates of rats. Expression of *iNOS* (Fig. 5A) in EAE animals treated during the symptomatic phase of EAE (EAE Cy HSCT II, EAE Cy HSCT Cy II) was considerably decreased in comparison to EAE non-treated animals (EAE). There were no significant differences in the expression of *iNOS* between other groups of animals.

There were no substantial differences observed in *Arg1* expression between the experimental groups of animals (Fig. 5B).

## Discussion

Multiple sclerosis is a human autoimmune disease, leading to neurodegeneration and progressing physical disability (Dobson and Giovannoni 2019). The primary immune reaction is evoked in the periphery, after the activation of autoreactive T cells. Next, after infiltration into CNS, T cells are restimulated by APCs, such as microglia, what start the inflammation directed to myelin antigens (Garg and Smith 2015). Both antigen-specific and innate mechanisms are involved in the pathomechanism of CNS inflammation. Resident microglia and infiltrating innate immune cells, after activation boosts the inflammation by releasing the pro-inflammatory cytokines and chemokines, participate in antigen presentation, phagocytosis, etc. (Garg and Smith 2015; Martin et al. 2016; Yadav et al. 2015). Those cells are also involved in the repair process after the inflammation resolution, by stimulating gliosis and remyelination (Bogie et al. 2014; Chu et al. 2018; Miron et al. 2013; Yadav et al. 2015).

The resident microglia have been shown to present both pro- and anti-inflammatory phenotypes – the M1 and M2 cells, respectively. Differentiation into M1 or M2 cells depends mostly on the surrounding environment (signals from other cells, inflammatory mediators, etc.). Over the active inflammation, M1 cells predominate, while during the resolution differentiation into M2 is observed (Bogie et al. 2014; Miron et al. 2013; Voet et al. 2019). It was also reported, that microglia may present the transitional phenotype, expressing both M1 and M2 markers (Bogie et al. 2014; Chu et al. 2019).

During present investigations, we analyzed the microglia phenotype in CNS of rats with evoked EAE and subjected to AHSCT. In treatment protocols used in MS patients, hematopoietic stem cell transplantation (HSCT) is preceded by immuno- or myeloablation, to remove the immune/autoreactive cells, which is done by administration of chemotherapeutic agent/agents (Martin et al. 2016; Muraro et al. 2017; Scolding et al. 2017).

In our experiments, rats were treated with high-dose of Cy, followed by AHSCT, and post-transplantation, low-dose of Cy. Treatment was applied according to two time schedules, during the pre-symptomatic or symptomatic phase of the EAE. Previously, we focused mostly on the disease course and symptoms observed in rats. We demonstrated, that such therapy is efficient in ameliorating the disease course, especially when applied during the symptomatic phase of the disease, and with additional, low-dose post-transplantation Cy (Kasarełło et al. 2021).

Histopathological analysis revealed the presence of inflammatory infiltrations in the spinal cords of animals with evoked EAE. Immunofluorescent examination of microglia phenotype in the cervical and thoracal spinal cord, and analysis of M1 and M2 markers levels in brain homogenates showed an overall consistent picture. M1 cell number and iNOS levels were similar in all experimental groups (except for a lowered number of M1 cells in control treated animals). The number of M2 cells and Arg1 levels were reduced after EAE evoking (a substantial drop of M2 cells number), and considerably increased when the therapy was applied in EAE rats, especially with an additional low-dose of Cy in the pre-symptomatic phase of EAE (M2 cells number), and both protocols in symptomatic phase of EAE (M2 cells number and Arg1 levels).

Experimental animals were sacrificed and tissues were collected in 21 DPI, during the remission of the disease symptoms (Mangano et al. 2010; Pitarokoili et al. 2017). Our results showed no changes in M1 microglia number, which is consistent with M1 cells pro-inflammatory activity, associated with relapse of the disease and the peak of symptoms. On the other hand, the remission of symptoms is related to the resolution of inflammation when differentiation towards the anti-inflammatory phenotype of immune cells occurs. Our data showed, that number of M2 cells is lower in EAE rats compared to control non-treated rats, manifesting the reduction of anti-inflammatory activity at this point, but the effect was reversed when the therapy was applied, especially in the symptomatic phase of EAE. This demonstrated, that used AHSCT protocols resulted in increased anti-inflammatory activity, leading to a decrease in EAE symptoms. This would be also beneficial, while M2 cells are associated with neurorepair processes (Chu et al. 2018).

Expression of *iNOS* and *Arg1* was also examined in brain homogenates and showed increased *iNOS* expression in non-treated EAE rats, and rats treated in the pre-symptomatic phase of the disease. This may point, that the inflammation is not fully quenched, with the potential to relapse. On the other hand, therapy applied during the symptomatic phase of the disease decreased the *iNOS* expression, possibly pointing to the remission of the inflammatory process. *Arg1* expression is equal in all experimental groups, what may predict the termination of anti-inflammatory activity.

Cy may affect not only the peripheral immune system but also by penetrating the blood–brain barrier, influence the resident immune cells (Atkins and Freedman 2013; Awad and Stüve 2009). Furthermore, those resident cells may be simply affected by a lowered number of infiltrating leukocytes, therefore, decreased levels of inflammatory mediators.

A few trials were conducted with the use of Cy in MS patients, with the conflicting outcome, but in general showing greater efficacy in patients with relapsing–remitting MS, where the inflammatory process is predominant over the degenerative one (Awad and Stüve 2009; Patti and Lo Fermo 2011; Schwartzman et al. 2009). This points to the importance of Cy immunomodulating activity.

The low dose of Cy at 40 mg/kg alone was tested in EAE Dark Agouti (DA) and Lewis rats with different outcomes depending on rat strain and day of Cy administration. In Lewis rats, Cy did not affect the overall clinical score of symptoms, only delaying the onset of symptoms, while in DA rats, Cy administration on the day of immunization and repeated on the day of symptoms onset almost totally inhibited the occurrence of the symptoms. Cy administration was accompanied by decreased inflammatory infiltrations in the spinal cord and decreased levels of pro-inflammatory cytokines (interferon γ, tumor necrosis factor α, and IL-12) in spinal cord homogenates (Mangano et al. 2010).

The use of cyclophosphamide is associated with side effects, which severity is related to the dose administered (Daikeler et al. 2012). Using high, immunoablative doses of Cy creates deep immunosuppression, which may be reversed by following reconstitution of the immune system by performing the transplantation of hematopoietic stem cells (Daikeler et al. 2012; Passweg et al. 2012). This method of myelo- or immunoablation followed by HSCT is recently approved in MS patients (Atkins et al. 2016; Burt et al. 2019; Cohen et al. 2019). Such therapy shows better outcomes in patients diagnosed with MS within the last 5 years, with active ongoing inflammation in the CNS (Atkins and Freedman 2013; Saccardi et al. 2006). There are many protocols of HSCT, among which those with the use of Cy are considered to be less toxic, but with a higher risk of disease relapse (Atkins and Freedman 2013).

In our experiments, we used the low toxicity protocol, where a high-dose of Cy is followed by AHSCT and next additional low-dose of Cy is administered, to eliminate the surviving autoreactive T cells (Luznik et al. 2012).

Results from our experiments clearly showed, that applied therapy emerged with differentiation of microglia towards M2, anti-inflammatory phenotype. This may be due to the direct effect of Cy on microglia, or indirect by decreasing the infiltration of the immune cells from the periphery. This benefits not only from the inhibition of inflammation but also from promoting the repair process. Microglia have been shown to influence the oligodendrocytes, which are responsible for the remyelination of demyelinated axons. Resting microglia and M2 cells support oligodendrocyte differentiation what is favorable for remyelination, while after activation, M1 microglia alter oligodendrocyte differentiation, and even stimulate its apoptosis (Cartier et al. 2014; Domingues et al. 2016; Miron et al. 2013).

There are some limitations of our study. It should be stated, that in the isolated from the euthanized animal tissue, except for the cells of the tissue itself, there will be some cells present in the blood vessels, thus the peripheral immune cells. Those will became the part of the cells subjected to analysis. Further, only a few microglial markers were used to assess the microglia phenotype. Our study was the preliminary research aiming to analyze the possible changes in the microglia phenotype, which reflects the immune activity in the CNS depending on the treatment protocol applied. Those had two variables; the time of the treatment application, and the presence/absence of an additional low dose of Cy. This resulted in great number of experimental groups to analyze. Nevertheless, results clearly showed that treatment during the symptomatic phase of the EAE is more beneficial, and further, more detailed analysis is needed.

To conclude, microglia, as the resident immune cells, participate in the inflammatory processes in the CNS accompanying neurodegenerative diseases such as MS. Differential phenotype of pro- or anti-inflammatory characteristic ensures the engagement of microglia both in the active phase of the disease, but also may contribute to repair and remyelination of surrounding tissue after the inflammation resolution. This underlines the importance of the therapies which influence the microglia and may change their phenotype towards M2, what would be beneficial not only for inhibiting the immune process but also for tissue regeneration.

## Data Availability

The datasets generated during and/or analyzed during the current study are available from the corresponding author on reasonable request.
